# Event-Triggered Dynamic Coverage Control for Multiple Stratospheric Airships

**DOI:** 10.3390/s22072734

**Published:** 2022-04-02

**Authors:** Yifei Zhang, Ming Zhu, Tian Chen

**Affiliations:** 1 School of Aeronautic Science and Engineering, Beihang University, Beijing 100191, China; zyifei@buaa.edu.cn; 2 Institute of Unmanned System, Beihang University, Beijing 100191, China; zhuming@buaa.edu.cn; 3 School of Electronic and Information Engineering, Beihang University, Beijing 100191, China

**Keywords:** dynamic coverage control, artificial dynamic potential field, event-triggered control

## Abstract

This article first investigates the dynamic coverage control problem for the multiple stratospheric airships (MSAs) system considering its practical application scenarios. A dynamic coverage control framework is put forward, in which the MSA system can be guided and controlled to fully cover the observation target region. Once a specific target is detected, the coverage target can be switched. First, the location information of the monitored target is predicted by an autoregressive model against processing delay. Second, the coverage control scheme consists of two layers: a novel potential field-based virtual control law to generate the desired velocity and angular velocity and an adaptive tracking controller to track them. In the virtual control law, a dynamic artificial potential field is introduced to adapt to the dynamic scenarios. In the tracking controller, which is combined with the adaptive control technique and the saturation compensator theory, the external disturbances and input saturation are addressed. Third, the event-triggered mechanism is designed to reduce the control frequency to prolong the actuator life. The simulation results are given to substantiate the capability of the proposed dynamic coverage control framework.

## 1. Introduction

The stratosphere (altitude ranges from 20 to 100 km) is the most peaceful layer of the earth’s atmosphere without the interference of the weather [1]. Owing to the rapid development of solar energy and material technology, the stratospheric airship has become a research hotspot in recent years thanks to its ability to perform tasks aloft or hovering around a specified area at the altitude of around 20 km [2]. Control research on the stratospheric airship includes path following [3,4], trajectory tracking [5,6], station keeping [7,8,9], formation tracking [10,11,12], and so on. As a high-altitude platform that can perform tasks in a fixed area for a long time, the demand for area coverage control is very strong. To expand the range of the coverage, the multiple stratospheric airships (MSAs) system, inheriting and enhancing the advantages of the single stratospheric airship, has emerged as an important research and application field [13]. Regarding the coverage control problem of the MSA system, the practical application characteristics, the recent research results of the MSA system, and the coverage control problem are analyzed in the following paragraphs.

First, several examples of recent research are cited for the control field of the MSA system as follows. In [10], the distributed event-triggered formation tracking problem of the MSA system with unknown nonlinearities was investigated. The adaptive fault-tolerant formation-containment control of the MSA system with input saturation was addressed in [11]. Based on [11], the problem of limited communication ranges was considered in [12]. To summarize, the research focus of the aforementioned works has been on the formation control of the MSA system in which the members of the MSA system are required to track a specified formation pattern. In this type of formation control, the control accuracy of the relative position is highlighted.

For the stratospheric airship and the MSA system, the main application scenarios are performing tasks such as reconnaissance surveillance, area monitoring, communication relay, and so forth [13]. To a certain extent, how to cover the target region to perform station keeping or to cover the target objective to track are more important than how to realize an accurate formation pattern. In addition, more characteristics of the MSA system should be considered as follows. As an LTA platform, the computing resources and actuator abilities are usually limited. Specifically, the computational efficiency of the controller and some actuator dynamic characteristics, such as input saturation, have to be properly considered. Moreover, the information data extraction of the observed or monitored target cannot be regarded as an ideal procession, and the processing delay should be taken into account.

The coverage control problem has been addressed by several works so far, whose main objective was to optimally place several mobile sensors to cover a target region [14,15,16,17,18,19,20,21,22,23,24,25,26]. However, for MSA systems, these works cannot be applied directly, due to the complex calculation of locational optimization, which challenges the computation ability of the airborne computer and is in conflicts with the energy system constraint. To cite some examples, a distributed dynamic area coverage algorithm based on reinforcement learning and a γ-information map for the multiagent system was investigated in [14]. In [15], low gain feedback was used to design distributed coverage control laws for the mobile sensors to minimize a coverage cost function to realize coverage control. In [16], Voronoi Partitions and optimal control method were utilized to design the coverage control law to realize the optimal partitioning for the moving coverage area for a group of autonomous mobile sensors. In [24], the Voronoi partitioning technique was adopted to realize the autonomous navigation of unmanned aerial vehicles (UAVs) for the surveillance of multiple moving ground targets. Based on [16], region coverage control problems under the situations of unknown density function [25] and uneven target distribution [26] were studied. In this paper, there is no need to define the optimal cost function, an artificial potential field (APF)-based coverage controller was designed, which ensures the coverage task is performed until the members of the MSA system end up in local minima.

Besides, another control problem that is closely related to coverage control is the circuit surveillance and monitoring [19,27,28,29], where the repetitive motion of the aerial vehicles is required. Unlike them, the coverage control problem of the MSA system is to design a mission-oriented formation controller to realize station-keeping control [7,8,9,30] for a group of stratospheric airships, which is likely a large-scale positioning control problem in [31], as the repetitive motion is unnecessary.

Input saturation is one of the important nonlinearities for engineering systems whose state-of-the-art approach is designed based on low-gain feedback [32]. This approach is first addressed based on an eigenstructure assignment algorithm, whereas the improved approaches are established by solving an algebraic Riccati equation (ARE) [33] and a parametric Lyapunov approach [34]. The objective of the event-triggered mechanism is to design a trigger condition to determine whether or not the control signal is executed [35,36,37], achieving the aim of reducing the control frequency of the actuators. Taking the above considerations into account, we properly solve the limitation problem of the actuator abilities to prolong the life of the actuators and realize precise control.

Motivated by the above aspects, we propose a dynamic coverage control framework for the MSA system with the consideration of its practical application characteristics to achieve switched region coverage control and moving target coverage control. The main contributions of the paper can be summarized as follows: (1) An efficient coverage control approach is designed based on the improved APF to realize the MSA system ability to cover the target region to monitor or observe the appearance of the specific moving target and, then, centralized coverage of the moving target if it appears. (2) To properly generate the potential field of the moving object to avoid chattering of the virtual law’s output, APF is improved into the artificial dynamic potential field (ADPF) by introducing velocity terms to modify the potential field. The proposed method has better performance than the traditional APF-based method. (3) A novel adaptive event-triggered controller is designed with an adaptive law and a saturation compensator to solve the problem of external disturbances and input saturation. The designed event-triggered mechanism can effectively reduce the control frequency of the actuators.

The rest of this paper is organized as follows. In Section 2, the preliminaries are provided. In Section 3, a dynamic coverage control framework for an MSA system is developed with the consideration of processing delay, unknown external disturbances, and input saturation, while mathematical proofs of the Lyapunov stability and the exclusion of Zeno behavior are given in Section 4. Finally, the simulation results and conclusion are given in Section 5 and Section 6, respectively.

## 2. Preliminaries

### 2.1. Notations and Lemmas

The following notations are adopted throughout this paper. R denotes the spaces for real numbers. $R^{n}$ denotes the *n*-dimensional Euclidean space, and $R^{m\times n}$ denotes the space of all real m×n matrices. Z denotes the spaces for integers, and $Z^{+}$ denotes the spaces for positive integers. $1^{n\times n}$ is a diagonal matrix whose elements are all 1. $∥\cdot ∥$ denotes the Euclidean norm or the Frobenius norm, $\left|\cdot \right|$ denotes the absolute value, i.e., for vector $x={\left[x_{1},x_{2},\ldots ,x_{n}\right]}^{T}\in R^{n}$, $\left|x\right|={\left[\left|x_{1}\right|,\left|x_{2}\right|,\cdots ,\left|x_{n}\right|\right]}^{T}$. $\lambda_{\max}\left(\cdot \right)$ denotes the largest eigenvalue, $\lambda_{\min}\left(\cdot \right)$ denotes the smallest eigenvalue. For vector $x={\left[x_{1},x_{2}\right]}^{T}$, $\arctan\left(x\right)=\arctan\left(\frac{x_{2}}{x_{1}}\right)$. For ∀x∈R or $\forall x\in R^{n}$, the saturation function is defined as

$$\mathrm{sat}\left(x\right)=\left\{\begin{array}{cc} x_{\max}, & x>x_{\max}\\ x, & x_{\min}\leq x\leq x_{\max}\\ x_{\min}, & x<x_{\min} \end{array}\right.$$

$$\mathrm{sat}\left(x\right)={\left[\mathrm{sat}\left(x_{1}\right),\mathrm{sat}\left(x_{2}\right),\cdots ,\mathrm{sat}\left(x_{n}\right)\right]}^{T}$$

**Lemma** **1.** *The equation 0≤|z|−ztanh(z/δ)≤0.2785δ holds for any δ>0 and z∈R [38].*

**Lemma** **2.** *For ∀a,b≥0, and p,q>0, satisfying 1/p+1/q=1, the inequality holds as $ab\leq \frac{a^{p}}{p}+\frac{b^{q}}{q}$ [39].*

### 2.2. System Description

**Assumption** **1.** *The stratospheric airships are all rigid bodies; thus, the aeroelastic effects of airships can be ignored.*

**Figure 1:**
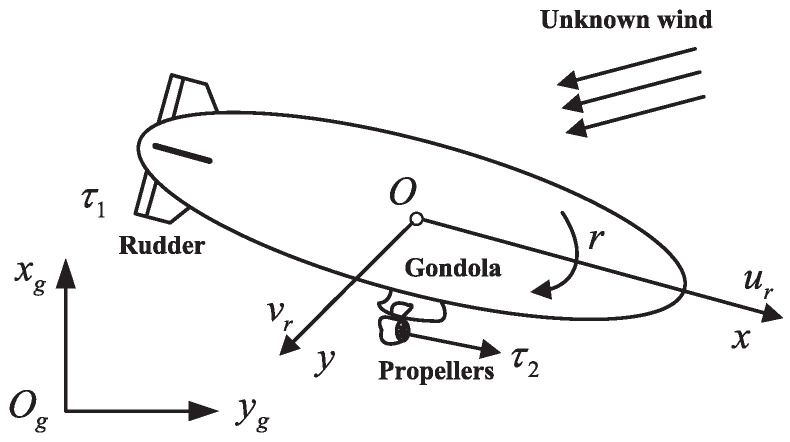
Structure of the stratospheric airship.

As shown in Figure 1, referring to [40], the kinematics and dynamics of member *i* of the MSAs can be expressed as

(1) $$\left\{\begin{array}{c} {\dot{\zeta }}_{i}=J_{i}\Theta_{i}\\ M_{i}{\dot{\Theta }}_{i}=N_{i}+f_{i}+\tau_{i}+d_{i} \end{array}\right.$$

where $\zeta_{i}={\left[\chi_{i},\psi_{i}\right]}^{T}$ denotes the vector of position $\chi_{i}={\left[x_{i},y_{i}\right]}^{T}$ and yaw angle $\psi_{i}$, $J_{i}=\mathrm{diag}\left[J_{p,i},1\right]$ denotes the coordinate transform matrix in which $J_{p,i}$ is defined as (2), $\Theta_{i}={\left[\Theta_{p,i},\Theta_{a,i}\right]}^{T}$ denotes the vector of velocities $\Theta_{p,i}={\left[u_{i},v_{i}\right]}^{T}$ and yaw angle velocity $\Theta_{a,i}$, $M_{i}$ denotes the generalized mass matrix as (3), $N_{i}$ denotes the vector of nonlinear coupling terms and aerodynamic forces and torque as (4), $\tau_{i}$ denotes the control vector, and $d_{i}$ denotes the vector of external disturbances.

**Remark** **1.** *The stratospheric airship usually stays at the same height thanks to the lift from the helium. Moreover, because the payload bay is fixed under its envelope, its roll angle and pitch angle are self-stable, similar to an inverted pendulum. Practically, the height, the roll angle, and the pitch angle are usually designed as uncontrollable in most overall designs. Therefore, the height, roll-angle, and pitch-angle control are all ignored in our controller design.*

**Assumption** **2.** *The positive real number ${\bar{d}}_{i}$ exists, which satisfies $∥d_{i}\left(t\right)∥\leq {\bar{d}}_{i}$, representing that the external disturbance $d_{i}\left(t\right)$ is unknown but bounded.*

(2) $$J_{p,i}=\left[\begin{array}{cc} \cos\psi_{i} & -\sin\psi_{i}\\ \sin\psi_{i} & \cos\psi_{i} \end{array}\right]$$

(3) $$M_{i}=\left[\begin{array}{ccc} m_{i}+\rho_{i}\nabla_{i}k_{1} & 0 & m_{i}y_{g,i}\\ 0 & m_{i}+\rho_{i}\nabla_{i}k_{2} & -m_{i}x_{g,i}\\ m_{i}y_{g,i} & -m_{i}x_{g,i} & I_{i,z}+\rho_{i}\nabla_{i}k_{3} \end{array}\right]$$

(4) $$N_{i}=\left[\begin{array}{c} \left(m_{i}+\rho_{i}\nabla_{i}k_{1}\right)v_{i}r_{i}+m_{i}x_{g,i}{r_{i}}^{2}+Q_{\infty ,i}C_{x,i}S_{ref,i}\\ -\left(m_{i}+\rho_{i}\nabla_{i}k_{2}\right)u_{i}r_{i}+m_{i}y_{g,i}{r_{i}}^{2}+Q_{\infty ,i}C_{y,i}S_{ref,i}\\ -m_{i}\left(x_{g,i}u_{i}r_{i}+y_{g,i}v_{i}r_{i}\right)+Q_{\infty ,i}C_{n,i}S_{ref,i}L_{ref,i} \end{array}\right]$$

### 2.3. APF Method

The APF method is utilized to guide MSAs to realize region coverage and moving target coverage. The APF, usually differentiated into the repulsive or the attractive potential fields, is constructed based on the relative distance between the APF generator and the APF receiver. Mathematically, in this paper, the resultant artificial potential field is composed of several different artificial potential fields denoted as potential functions. For each particular potential function with its potential function is $P_{k}$, its potential force can be obtained as

(5) $$F_{k}\left(d_{k}\right)=\frac{\partial P_{k}}{\partial d_{k}}.$$

where $d_{k}$ denotes the relative distance. Therefore, the resultant potential force, consisting of *n* potential functions, can be obtained as

(6) $$\begin{array}{cc} F & =\sum_{k=1}^{n}F_{k}\left(d_{k}\right)\\ \; & =\sum_{k=1}^{n}\frac{\partial P_{k}}{\partial d_{k}} \end{array}$$

### 2.4. Control Objective

**Figure 2:**
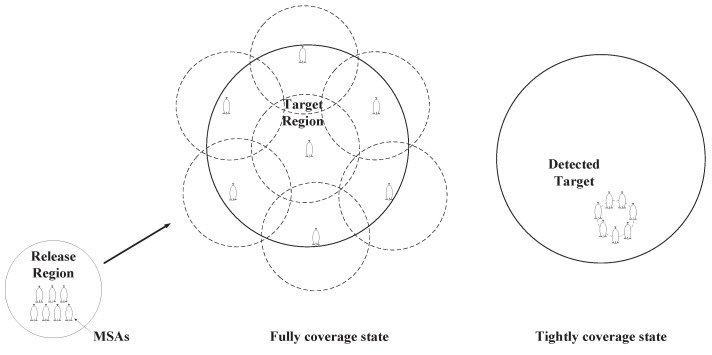
Depiction of the region-coverage mission.

As shown in Figure 2, the objective of this paper is to design a dynamic coverage control framework for the MSA system to accomplish the following tasks: full coverage state: the MSA system can be guided and controlled to reach the target region and fully cover it to monitor or observe; tight coverage state: the MSA system can be guided and controlled to cover the detected moving target and track it. Specifically, the objectives of the designed control framework are as follows:

(1) Several potential functions are designed based on the demands of the dynamic coverage mission;
(2) Virtual control laws $\Theta_{p,i}^{d}$ and $\Theta_{a,i}^{d}$ are designed to guarantee that all members of the MSA system can be guided to the global minimum point, to end up covering the target region or detecting the moving target;
(3) The influence of location information processing delay can be eliminated by the AR model;
(4) The problems of external disturbances and input saturation can be solved by the control scheme; and
(5) The control frequency of the actuator can be reduced by the control scheme.

## 3. Main Results

The main results are presented in this section and are summarized as follows.

(1) The APF method is introduced to provide a guidance method to guide airships to achieve the missions of dynamic coverage, in which the MSA system is supposed to cover the target region to monitor or observe for the appearance of the specific moving target, and then attain centralized coverage of the moving target if it appears. Considering that some APF generators are moving, the APF is improved to be the artificial dynamic potential field (ADPF) by introducing the velocity term into it to better formulate the potential field;
(2) The location of the moving target is forecast online by means of an AR-based position predictor, considering the processing time delay of the observation results of these moving targets;
(3) To meet the aim of dynamic coverage, an ADPF-based event-triggered adaptive controller is designed, considering the external disturbances and the input saturation. Towards this end, rigorous theoretical analysis for the Lyapunov stability and the exclusion of Zeno behavior are provided.

### 3.1. Position Prediction of Target

Considering the actual complex application scenario that the ability of the telemetry equipment is limited, the location determination of the target will have a time delay to some extent. Generally, the length of this time delay, only depending on the ability of the telemetry equipment, is fixed, which can be obtained by the measurement in a ground hardware-in-loop experiment. To estimate the current location of the target based on history data, the AR model is established as follows:

(7) $$d\left(t\right)=\sum_{i=1}^{n}a_{i}d\left(t-i\right)+e\left(t\right)$$

where *n* is the order of model, $a_{i}$ is the coefficient of model, and e(t) is the prediction error.

A vector-form AR prediction model can be expressed as

(8) d=DA+e

where $d={\left[d\left(n+1\right),d\left(n+2\right),\ldots ,d\left(K\right)\right]}^{T}$ with *K* as the sampled amount of the past location information, $A={\left[a_{1},a_{2},\ldots ,a_{k}\right]}^{T}$, $e={\left[e(n+1),e(n+2),\ldots ,e(K)\right]}^{T}$, and

(9) $$D=\left[\begin{array}{cccc} d(n) & d(n-1) & \cdots & d(1)\\ d(n+1) & d(n) & \cdots & d(2)\\ \ldots & \cdots & \cdots & \cdots \\ d(K-1) & d(K-2) & \cdots & d(K-n) \end{array}\right]$$

Based on the least square method, the coefficient vector A can be estimated as

(10) $$\hat{A}\left(K\right)=P\left(K\right)Q\left(K\right)$$

where the factor matrices $P\left(K\right)={\left[\sum_{i=n+1}^{K}D^{T}\left(i\right)D\left(i\right)\right]}^{-1}$ and $Q\left(K\right)=\sum_{i=n+1}^{K}D^{T}\left(i\right)d\left(i\right)$ can be derived by minimizing J=(d−${\left.D\hat{A}\right)}^{T}\left(d-D\hat{A}\right)$.

The optimal order $n^{*}$ is specified according to the following Bayesian Information Criterion (BIC) method:

(11) $$BIC\left(n,K\right)=\ln\left(\frac{\sum_{i=n+1}^{K}{\left[d\left(t\right)-A\left(t\right)D^{T}\left(t\right)\right]}^{2}}{K-n}\right)+\frac{n\ln K}{K-n}$$

(12) $$n^{*}=\underset{n}{\arg\min}\mathrm{BIC}\left(n,K\right)$$

To avoid distortion of the AR model, the max order is normally set as K/3.

Referring to [41], the realtime position can be predicted by

(13) $$d\left(N+\delta \right)=\left\{\begin{array}{cc} \sum_{i=1}^{n^{*}}{\hat{a}}_{i}d\left(N+\delta -i\right), & \delta =1\\ \sum_{i=1}^{\delta }{\hat{a}}_{i}d\left(N+\delta -i\right)+\sum_{i=\delta +1}^{n^{*}}{\hat{a}}_{i}\hat{d}\left(N+\delta -i\right), & 1<\delta \leq n^{*}\\ \sum_{i=1}^{n^{*}}{\hat{a}}_{i}\hat{d}\left(N+\delta -i\right), & \delta >n^{*} \end{array}\right.$$

where δ is the prediction step related to the length of time delay.

**Remark** **2.** *Consequently, the realtime velocity of the target can be obtained by differentiating the realtime position information.*

### 3.2. Dynamic Coverage Potential Field Design

The designed ADPF consists of four potential functions as follows: a region attractive potential function denoted as $P_{r,i}^{a}$ for the target region to guide airships to reach the target region, a mutual repulsive potential function denoted as $P_{m,i}^{r}$ to guide airships to fully cover the target region, a mutual attractive potential function denoted as $P_{m,i}^{a}$ for communication connectivity, and an attractive potential function denoted as $P_{t,i}^{a}$ for the moving target to guide the MSA system to track and cover it. The above four potential functions are designed in Section 3.2.2, Section 3.2.3, Section 3.2.4 and Section 3.2.5.

#### 3.2.1. Improved Dynamic Potential Field Design

In this paper, the artificial potential field is used to calculate the artificial potential force generated by the moving objects, not the static objects. For the traditional artificial potential field as $P_{k}$, the potential force is generated by the static objects and is obtained as (5) without the reflection of the relative velocity. In our problem to be studied, the whole system is dynamic; however, it is necessary to introduce the velocity terms into the utilization of the APF and turn the APF into the artificial dynamic potential field (ADPF). Define the relative distance as $d_{k}=\chi_{t}-\chi_{i}$, where $\chi_{i}$ is the position of the airship member *i*, and $\chi_{t}={\left[x_{t},y_{t}\right]}^{T}$ is the center of the moving object; the Equation (5) can be expanded as

(14) $$\begin{array}{cc} F_{k}\left(d_{k}\right) & =\frac{\partial P_{k}}{\partial d_{k}}+\eta \frac{\partial P_{k}}{\partial d_{k}}\tanh\left({\dot{d}}_{k}\right)\mathrm{sign}\left(\frac{\partial P_{k}}{\partial d_{k}}\right)\\ \; & =\frac{\partial P_{k}}{\partial d_{k}}\left(1+\eta \tanh\left({\dot{x}}_{i,t}\cos\psi_{i,t}+{\dot{y}}_{i,t}\sin\psi_{i,t}\right)\mathrm{sign}\left(\frac{\partial P_{k}}{\partial d_{k}}\right)\right) \end{array}$$

In (14), with η∈(0,1); $\psi_{i,t}$, ${\dot{x}}_{i,t}$ and ${\dot{y}}_{i,t}$ are defined as (15).

(15) $$\left\{\begin{array}{cc} {\dot{x}}_{i,t} & ={\dot{x}}_{t}-{\dot{x}}_{i}\\ {\dot{y}}_{i,t} & ={\dot{y}}_{t}-{\dot{y}}_{i}\\ \psi_{i,t} & =\psi_{t}-\psi_{i} \end{array}\right.$$

**Remark** **3.** *The value range of the parameter η is designed to be (0,1) to guarantee that this design will not change the direction of the generated potential force.*

**Figure 3:**
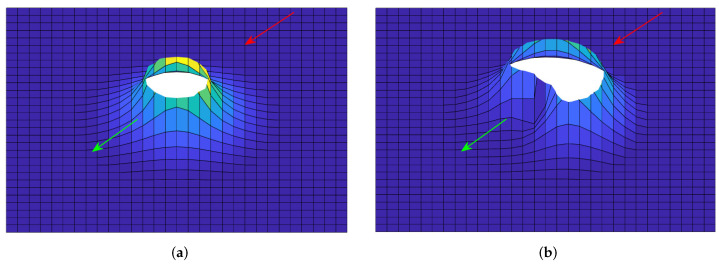
Comparison between APF and ADPF for a repulsive potential field. (**a**) APF, (**b**) ADPF.

**Figure 4:**
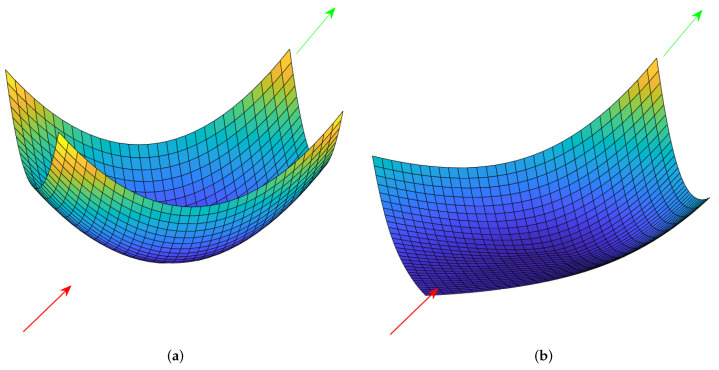
Comparison between APF and ADPF for an attractive potential field. (**a**) APF, (**b**) ADPF.

In Figure 3 and Figure 4, the comparisons between APF and ADPF for a repulsive potential field and an attractive potential field are given. The generator of the potential field is located in the center in each figure in Figure 3 and Figure 4. The arrow denotes the direction of the relative velocity of the potential field receiver compared with the generator of the potential field. The red arrow denotes that the direction of the relative velocity is toward the potential generator, and the green arrow denotes that the direction of the relative velocity is away from the potential generator. The potential fields were generated under different directions of the relative movements with the same relative velocity, from which the conclusions can be obtained as follows: for the repulsive potential field, if the direction of relative movement is away from the potential generator, the potential is lower than that if the direction of relative movement is toward the potential generator; for the attractive potential field, if the direction of relative movement is away from the potential generator, the potential is higher than that if the direction of relative movement is toward the potential generator. Apparently, this adaptive design can help calculate a more reasonable potential force according to the relative movement to realize the aim of avoiding location oscillations.

#### 3.2.2. Region Attractive Potential Function

Define the relative distance from the airship member *i* to the target region as $d_{i}^{a}=\chi_{a}-\chi_{i}$, where $\chi_{i}$ is the position of the airship member *i*, and $\chi_{a}={\left[x_{a},y_{a}\right]}^{T}$ is the center of the target region; then, the region attractive potential function of target region for airship member *i* is designed as $P_{r,i}^{a}=\frac{1}{2}{\left(\max\left\{0,{∥d_{i}^{a}∥}^{2}-R^{2}\right\}\right)}^{2}$ where *R* is the radius of the target region. In addition, the corresponding potential force can be obtained as $F_{r,i}^{a}\left(\chi_{i}\right)=-2d_{i}^{a}\left(\max\left\{0,{∥d_{i}^{a}∥}^{2}-R^{2}\right\}\right)$.

#### 3.2.3. Mutual Repulsive Potential Function

For airship member *i*, $d_{i}^{j}=\chi_{j}-\chi_{i}$ is the distance from the airship member *i* to the airship member *j*, as $\chi_{j}$ denotes the position of airship member *j*, and $\rho^{r}$ is the desired relative distance between *i* and *j*; then, the mutual repulsive potential field of *j* for *i* is designed as $P_{r,m}\left(d_{i}^{j}\right)=\frac{1}{2}{\left(\max\left\{0,-\ln\left({∥d_{i}^{j}∥}^{2}\right)+\ln\left({\rho^{r}}^{2}\right)\right\}\right)}^{2}$ and the corresponding potential force can be obtained as $F_{r,m}\left(d_{i}^{j}\right)=\frac{2\theta_{r,m}d_{i}^{j}}{{∥d_{i}^{j}∥}^{2}}\left(\max\left\{0,-\ln\left({∥d_{i}^{j}∥}^{2}\right)+\ln\left({\rho^{r}}^{2}\right)\right\}\right)$ where $\theta_{r,m}=1+\eta_{r,m}\tanh\left({\dot{x}}_{i,j}\cos\psi_{i,j}+{\dot{y}}_{i,j}\sin\psi_{i,j}\right)\mathrm{sign}\left(d_{i}^{j}\right)>0$ with $\eta_{r,m}\in \left(0,1\right)$.

The total mutual repulsive potential function $P_{m,i}^{r}$ for *i* is $P_{m,i}^{r}=\sum_{j=0}^{n-1}P_{r,m}\left(d_{i}^{j}\right)$ and the total mutual artificial repulsive force is $F_{m,i}^{r}\left(\chi_{i}\right)=\sum_{j=0}^{n-1}F_{r,m}\left(d_{i}^{j}\right)$.

#### 3.2.4. Mutual Attractive Potential Function

The mutual attractive potential field $P_{m,i}^{a}$ of *j* for *i* is designed as $P_{a,m}\left(d_{i}^{j}\right)=\frac{1}{2}\left(\max\right.$${\left.\left\{0,\ln\left({∥d_{i}^{j}∥}^{2}\right)-\ln\left({\rho^{a}}^{2}\right)\right\}\right)}^{2}$ and the corresponding artificial attractive force is $F_{a,m}\left(d_{i}^{j}\right)=-\frac{2\theta_{r,m}d_{i}^{j}}{{∥d_{i}^{j}∥}^{2}}\left(\max\left\{0,\ln\left({∥d_{i}^{j}∥}^{2}\right)-\ln\left({\rho^{a}}^{2}\right)\right\}\right)$ in which $\theta_{a,m}=1+\eta_{a,m}\tanh\left({\dot{x}}_{i,j}\cos\psi_{i,j}+{\dot{y}}_{i,j}\right.$$\left.\sin\psi_{i,j}\right)\mathrm{sign}\left(-d_{i}^{j}\right)>0$ with $\eta_{a,m}\in \left(0,1\right)$.

The total mutual attractive potential function $P_{m,i}^{a}$ for *i* is $P_{m,i}^{a}\left(\chi_{i}\right)=\sum_{j=0}^{n-1}P_{a,m}\left(d_{i}^{j}\right)$ and the total mutual artificial attractive force is $F_{m,i}^{a}\left(\chi_{i}\right)=\sum_{j=0}^{n-1}F_{a,m}\left(d_{i}^{j}\right)$

#### 3.2.5. Moving Target Attractive Potential Function

For airship member *i*, $d_{i}^{t}=\chi_{t}-\chi_{i}$ is the distance from the airship member *i* to the moving target, as $\chi_{t}$ denotes the position of the moving target, and *r* is the radius of the moving target; then, the moving target following potential field for *i* is designed as $P_{t,i}^{a}\left(d_{i}^{t}\right)=\frac{1}{2}{\left(\max\left\{0,-\ln\left({∥d_{i}^{t}∥}^{2}\right)+\ln\left(r^{2}\right)\right\}\right)}^{2}$ and the corresponding potential force can be obtained as $F_{t,i}^{a}\left(d_{i}^{t}\right)=-\frac{2\theta_{a,t}d_{i}^{t}}{{∥d_{i}^{t}∥}^{2}}\left(\max\left\{0,-\ln\left({∥d_{i}^{t}∥}^{2}\right)+\ln\left(r^{2}\right)\right\}\right)$ where $\theta_{a,t}=1+\eta_{a,t}\tanh\left({\dot{x}}_{i,t}\cos\psi_{i,t}+{\dot{y}}_{i,t}\sin\psi_{i,t}\right)\mathrm{sign}\left(-d_{i}^{t}\right)>0$ with $\eta_{a,t}\in \left(0,1\right)$.

### 3.3. Virtual Control Law Design

Based on the designed ADPF in Section 3.2, the virtual position control law(16) is designed to generate the desired velocity for MSAs member *i* to track to realize the control objective.

(16) $$\begin{array}{cc} \Theta_{p,i}^{d} & =-{J_{p,i}}^{-1}\left(k_{p}\left(k_{r,a}F_{r,i}^{a}+k_{m,a}F_{m,i}^{a}+k_{m,r}F_{m,i}^{r}+k_{a,t}\phi F_{t,i}^{a}\right)\right) \end{array}$$

where $k_{p}$, $k_{r,a}$, $k_{r,r}$, $k_{m,a}$, and $k_{m,r}\in R^{+}$ are control parameters; ϕ is the triggering flag, if the value of the triggering flag is determined by

(17) $$\phi =\left\{\begin{array}{cc} 1, & \mathrm{the}\;\mathrm{moving}\;\mathrm{target}\;\mathrm{is}\;\mathrm{detected}\\ 0, & \mathrm{the}\;\mathrm{moving}\;\mathrm{target}\;\mathrm{isn}\text{’}t\;\mathrm{detected} \end{array}\right.$$

As the desired yaw angle is the desired moving direction, in other words, the direction of the desired velocity $\Theta_{p,i}^{d}$, thus the tracking error of the current yaw angle, can be defined as

(18) $$\xi_{a,i}=\psi_{i}-\arctan\left(\Theta_{p,i}^{d}\right)$$

Based on tracking error (18), the virtual yaw-angle control law is designed as

(19) $$\Theta_{a,i}^{d}=-k_{\psi }\left(\psi_{i}-\arctan\left(\Theta_{p,i}^{d}\right)\right)$$

where $k_{\psi }\in R^{+}$ is the chosen control parameter.

Consider a Lyapunov candidate for $\Theta_{p,i}^{d}$ as (20).

(20) $$V_{1}=\sum_{i=1}^{n}\left(k_{a,r}P_{r,i}^{a}+\frac{\vartheta_{r,m}}{2}k_{a,m}P_{m,i}^{a}+\frac{\vartheta_{a,m}}{2}k_{r,m}P_{m,i}^{r}+\frac{\vartheta_{a,t}}{2}\phi k_{a,t}P_{t,i}^{a}\right)>0$$

The derivative of (20) can be obtained as

(21) $$\begin{array}{cc} {\dot{V}}_{1}= & \sum_{i=1}^{n}{\dot{\chi }}_{i}^{T}\left(k_{a,r}F_{r,i}^{a}+k_{a,m}F_{m,i}^{a}+k_{r,m}F_{m,i}^{r}+\phi k_{a,t}F_{t,i}^{a}\right)\\ \leq & -\sum_{i=1}^{n}{\left(k_{p}\left(k_{r,a}F_{r,i}^{a}+k_{m,a}F_{m,i}^{a}+k_{m,r}F_{m,i}^{r}+k_{a,t}\phi F_{t,i}^{a}\right)\right)}^{T}\\ \; & \left(k_{a,r}F_{r,i}^{a}+k_{a,m}F_{m,i}^{a}+k_{r,m}F_{m,i}^{r}+\phi k_{a,t}F_{t,i}^{a}\right)\\ \leq & -\sigma_{1}V_{1} \end{array}$$

where $\sigma_{1}>0$.

Consider another Lyapunov candidate for $\Theta_{a,i}^{d}$ as (22).

(22) $$\begin{array}{c} V_{2}=\frac{1}{2}\xi_{a,i}^{2} \end{array}$$

Its derivative can be obtained as

(23) $$\begin{array}{cc} {\dot{V}}_{2} & =\xi_{a,i}{\dot{\xi }}_{a,i}\\ \; & =-k_{\psi }{\left(\psi_{i}-\arctan\left(\Theta_{p,i}^{d}\right)\right)}^{2}\\ \; & =-2k_{\psi }V_{2} \end{array}$$

### 3.4. Event-Triggered Adaptive Tracking Controller Design

Define the tracking error of the desired velocity/angular velocity $\Theta_{i}={\left[{\Theta_{p,i}^{d}}^{T},\Theta_{a,i}^{d}\right]}^{T}$ and the real velocity/angular velocity $\Theta_{i}^{d}$ as

(24) $$\xi_{\Theta ,i}=\Theta_{i}-\Theta_{i}^{d}$$

Differentiate (24) with respect to time as

(25) $${\dot{\xi }}_{\Theta ,i}={\dot{\Theta }}_{i}-{\hat{\dot{\Theta }}}_{i}^{d}-{\tilde{\dot{\Theta }}}_{i}^{d}$$

where ${\hat{\dot{\Theta }}}_{i}^{d}$ is the estimate value of ${\dot{\Theta }}_{i}^{d}$ obtained by a second-order filter, which is used to cope with the intricate computation problem of differentiating ${\dot{\Theta }}_{i}^{d}$ [42]. As the estimate error of ${\dot{\Theta }}_{i}^{d}$ is defined as ${\tilde{\dot{\Theta }}}_{i}^{d}={\dot{\Theta }}_{i}^{d}-{\hat{\dot{\Theta }}}_{i}^{d}$, the derivative of (18) can be obtained as (19).

Then, (19) can be expanded as

(26) $$\begin{array}{cc} {\dot{\xi }}_{\Theta ,i} & ={\dot{\Theta }}_{i}-{\dot{\Theta }}_{i}^{d}\\ \; & =M_{i}^{-1}\left(N_{i}+\tau_{i}+d_{i}\right)-{\hat{\dot{\Theta }}}_{i}^{d}-{\tilde{\dot{\Theta }}}_{i}^{d}\\ \; & =M_{i}^{-1}\left(N_{i}+\tau_{i}+d_{total,i}\right)-{\hat{\dot{\Theta }}}_{i}^{d} \end{array}$$

where $d_{total,i}=d_{i}-M_{i}{\tilde{\dot{\Theta }}}_{i}^{d}$ is the nominal disturbance.

**Remark** **4.** *The estimate error ${\tilde{\dot{\Theta }}}_{i}^{d}$ of second-order filter is bounded in a small neighborhood of zero [42]. Derived from Assumption 2, the external disturbances $d_{i}$ are bounded. Therefore, $d_{total,i}$ is a bounded vector satisfying $d_{total,i}\leq {\bar{d}}_{total,i}$.*

To reduce the influence of the nominal disturbance $d_{total,i}$, an adaptive law is designed as

(27) $$\dot{{\hat{d}}_{i}}=\alpha \left(\xi_{\Theta ,i}-\beta {\hat{d}}_{i}\right)$$

where both positive matrices α and β$\in R^{3\times 3}$ are adaptation parameters to be chosen.

Considering the event-triggered mechanism designed afterwards, the event-triggered adaptive tracking controller is designed as

(28) $$\tau_{i}=-M_{i}\left(K_{\Theta }\left(\xi_{\Theta ,i}-\zeta_{i}\right)+K_{d}{\hat{d}}_{i}-\bar{\kappa }+{\hat{\dot{\Theta }}}_{i}^{d}\right)-N_{i}$$

where $\zeta_{i}$ is the output of the saturation compensator designed afterwards, ${\bar{\kappa }}_{i}=\mathrm{diag}[\kappa_{\lambda }\tanh$$\left(\frac{\kappa_{\lambda }\xi_{\Theta ,i,\lambda }}{k_{\kappa ,\lambda }})\right]$ and positive matrices $\bar{\kappa }=\mathrm{diag}\left[\bar{\kappa }\right]$,$K_{\kappa }=\mathrm{diag}\left[k_{\kappa ,\lambda }\right]$,$K_{\Theta }$, $K_{d}$$\in R^{3\times 3}$ all are controller parameters to be chosen.

To reduce the influence of input saturation, the saturation compensator $\zeta_{i}$ is designed as

(29) $${\dot{\zeta }}_{i}=-K_{\zeta }\zeta_{i}+\Delta \tau_{i}$$

where $\Delta \tau_{i}=\tau_{i}^{*}-\tau_{i}$, and $\tau_{i}^{*}=\mathrm{sat}\left(\tau_{i}\right)$ is the bounded control input, and $K_{\zeta }=\mathrm{diag}\left[k_{\zeta ,\lambda }\right]$ is the compensator parameter to be chosen.

The event-triggered mechanism is designed as

(30) $$\left\{\begin{array}{c} u_{i}=\tau_{i}\left(t_{m}\right),t\in \left[t_{m},t_{m+1}\right)\\ t_{m+1}=\inf\{t\in R|\exists \left|e_{i,\lambda }\left(t\right)\right|\geq \gamma_{\lambda }\tanh|u_{i,\lambda }\left|+\kappa_{\lambda },\lambda =1,2,3\right\} \end{array}\right.$$

where $e_{i}=\tau_{i}-u_{i}$; the positive matrices $\gamma_{\lambda }=\mathrm{diag}\left[\gamma_{\lambda }\right]$$\in R^{3\times 3}$,$\kappa =\mathrm{diag}[\kappa_{\lambda }]$ are the triggering parameter matrices satisfying $\kappa_{\lambda }>\gamma_{\lambda }+\kappa_{\lambda }$.

Consider a Lyapunov candidate

$$V_{3}=\frac{1}{2}\xi_{\Theta ,i}^{T}\xi_{\Theta ,i}+\frac{1}{2}{\hat{d}}_{i}^{T}{\hat{d}}_{i}+\frac{1}{2}\zeta_{i}^{T}\zeta_{i}$$

Its derivative can be obtained as

(31) $$\begin{array}{cc} {\dot{V}}_{3} & =\xi_{\Theta ,i}^{T}{\dot{\xi }}_{\Theta ,i}+{\hat{d}}_{i}^{T}\dot{{\hat{d}}_{i}}+\zeta_{i}^{T}{\dot{\zeta }}_{i}\\ \; & =\xi_{\Theta ,i}^{T}\left({M_{i}}^{-1}\left(N_{i}+u_{i}+d_{total,i}\right)-{\hat{\dot{\Theta }}}_{i}^{d}\right)+{\hat{d}}_{i}^{T}\left(\alpha \left(\xi_{\Theta ,i}-\beta {\hat{d}}_{i}\right)\right)+\zeta_{i}^{T}\left(-K_{\zeta }\zeta_{i}+\Delta \tau_{i}\right) \end{array}$$

It is known from (28) that

(32) $$\begin{array}{cc} |u_{i,\lambda }-\tau_{i,\lambda }| & <\gamma_{\lambda }\tanh\left|u_{i,\lambda }\right|+\kappa_{\lambda }\\ \; & <\gamma_{\lambda }+\kappa_{\lambda }, \end{array}$$

for $t\in \left[t_{m},t_{m+1}\right)$ such that *u* can be written as

(33) $$u_{i}=\tau_{i}\left(t\right)-\left(\gamma +\kappa \right)\iota_{i}\left(t\right)$$

where $\iota_{i,\lambda }\left(t\right)=\frac{\tau_{\lambda }\left(t\right)-u_{\lambda }}{\gamma_{\lambda }+\kappa_{\lambda }}$ is continuous and $|\iota_{i,\lambda }\left(t\right))|<1$.

Substituting (32) into (30) yields

(34) $$\begin{array}{cc} {\dot{V}}_{3}= & \xi_{\Theta ,i}^{T}\left(-K_{\Theta }\left(\xi_{\Theta ,i}-\zeta_{i}\right)-K_{d}{\hat{d}}_{i}+\bar{\kappa }-\left(\gamma +\kappa \right)\iota_{i}\left(t\right)\right)+{\hat{d}}_{i}^{T}\left(\alpha \left(\xi_{\Theta ,i}-\beta {\hat{d}}_{i}\right)\right)\\ \; & +\zeta_{i}^{T}\left(-K_{\zeta }\zeta_{i}+\Delta \tau_{i}\right)+{M_{i}}^{-1}\xi_{\Theta ,i}^{T}d_{total,i} \end{array}$$

Based on Lemma 1, it follows that

(35) $$\begin{array}{cc} \; & -\xi_{\Theta ,\lambda }\left(\left(\gamma_{\lambda }+\kappa_{\lambda }\right)\iota_{\lambda }+\kappa_{\lambda }\tanh\left(\frac{\kappa_{\lambda }\xi_{\Theta ,\lambda }}{K_{\kappa ,\lambda }}\right)\right)\\ \; & \leq \left(\left|\xi_{\Theta ,\lambda }\left(\gamma_{\lambda }+\kappa_{\lambda }\right)\right|-\kappa_{\lambda }\xi_{\Theta ,\lambda }\tanh\left(\frac{\kappa_{\lambda }\xi_{\Theta ,\lambda }}{K_{\kappa ,\lambda }}\right)\right)\\ \; & \leq \left(\left|\kappa_{\lambda }\xi_{\Theta ,\lambda }\right|-\kappa_{\lambda }\xi_{\Theta ,\lambda }\tanh\left(\frac{\kappa_{\lambda }\xi_{\Theta ,\lambda }}{K_{\kappa ,\lambda }}\right)\right)\\ \; & \leq 0.2785K_{\kappa ,\lambda }. \end{array}$$

Substituting (35) into (34) and using Young’s inequality in Lemma 2 with suitable $\varepsilon_{1},\varepsilon_{2},\varepsilon_{3},\varepsilon_{4}>0$ yields

(36) $$\begin{array}{cc} {\dot{V}}_{3}\leq & -K_{\Theta }\xi_{\Theta ,i}^{T}\xi_{\Theta ,i}-\alpha \beta {\hat{d}}_{i}^{T}{\hat{d}}_{i}-K_{\zeta }\zeta_{i}^{T}\zeta_{i}+K_{\Theta }\xi_{\Theta ,i}^{T}\zeta_{i}-K_{d}\xi_{\Theta ,i}^{T}{\hat{d}}_{i}+\alpha {\hat{d}}_{i}^{T}\xi_{\Theta ,i}+\zeta_{i}^{T}\Delta \tau_{i}\\ \; & +{M_{i}}^{-1}\xi_{\Theta ,i}^{T}d_{total,i}+0.2785\lambda_{max}\left(K_{\kappa }\right)\\ \leq & -K_{\Theta }\xi_{\Theta ,i}^{T}\xi_{\Theta ,i}-\alpha \beta {\hat{d}}_{i}^{T}{\hat{d}}_{i}-K_{\zeta }\zeta_{i}^{T}\zeta_{i}+\lambda_{\max}\left(K_{\Theta }\right)∥\xi_{\Theta ,i}∥∥\zeta_{i}∥+\lambda_{\max}\left(\alpha -K_{d}\right)∥\xi_{\Theta ,i}∥∥{\hat{d}}_{i}∥\\ \; & +∥\zeta_{i}∥∥\Delta \tau_{i}∥+\lambda_{\max}\left({M_{i}}^{-1}\right)∥\xi_{\Theta ,i}∥{\bar{d}}_{total,i}+0.2785\lambda_{max}\left(K_{\kappa }\right)\\ \leq & -\lambda_{\min}\left(K_{\Theta }\right)\xi_{\Theta ,i}^{T}\xi_{\Theta ,i}-\lambda_{\min}\left(\alpha \beta \right){\hat{d}}_{i}^{T}{\hat{d}}_{i}-\lambda_{\min}\left(K_{\zeta }\right)\zeta_{i}^{T}\zeta_{i}+\lambda_{\max}\left(K_{\Theta }\right)(\frac{\varepsilon_{1}}{2}{∥\xi_{\Theta ,i}∥}^{2}\\ \; & +\frac{1}{2\varepsilon_{1}}{∥\zeta_{i}∥}^{2})+\lambda_{\max}\left(\alpha -K_{d}\right)\left(\frac{\varepsilon_{2}}{2}{∥\xi_{\Theta ,i}∥}^{2}+\frac{1}{2\varepsilon_{2}}{∥{\hat{d}}_{i}∥}^{2}\right)+\frac{\varepsilon_{3}}{2}{∥\zeta_{i}∥}^{2}+\frac{1}{2\varepsilon_{3}}{∥\Delta \tau_{i}∥}^{2}\\ \; & +\lambda_{\max}\left({M_{i}}^{-1}\right)\left(\frac{\varepsilon_{4}}{2}{∥\xi_{\Theta ,i}∥}^{2}+\frac{1}{2\varepsilon_{4}}{{\bar{d}}_{total,i}}^{2}\right)+0.2785\lambda_{max}\left(K_{\kappa }\right)\\ \leq & -\left(\lambda_{\min}\left(K_{\Theta }\right)-\frac{\varepsilon_{1}}{2}\lambda_{\max}\left(K_{\Theta }\right)-\frac{\varepsilon_{2}}{2}\lambda_{\max}\left(\alpha -K_{d}\right)-\frac{\varepsilon_{4}}{2}\lambda_{\max}\left({M_{i}}^{-1}\right)\right)\xi_{\Theta ,i}^{T}\xi_{\Theta ,i}\\ \; & -\left(\lambda_{\min}\left(\alpha \beta \right)-\frac{1}{2\varepsilon_{2}}\lambda_{\max}\left(\alpha -K_{d}\right)\right){\hat{d}}_{i}^{T}{\hat{d}}_{i}-\left(\lambda_{\min}\left(K_{\zeta }\right)-\frac{1}{2\varepsilon_{2}}\lambda_{\max}\left(K_{\Theta }\right)-\frac{\varepsilon_{3}}{2}\right)\zeta_{i}^{T}\zeta_{i}\\ \; & +\frac{1}{2\varepsilon_{3}}{∥\Delta \tau_{i}∥}^{2}+\frac{1}{2\varepsilon_{4}}\lambda_{\max}\left({M_{i}}^{-1}\right){{\bar{d}}_{total,i}}^{2}+0.2785\lambda_{max}\left(K_{\kappa }\right)\\ \leq & -\sigma_{3}V_{3}+C \end{array}$$

where $\sigma_{3}=\min\left\{2\lambda_{\min}\left(K_{\Theta }\right)-\varepsilon_{1}\lambda_{\max}\left(K_{\Theta }\right)-\varepsilon_{2}\lambda_{\max}\left(\alpha -K_{d}\right)-\varepsilon_{4}\lambda_{\max}\left({M_{i}}^{-1}\right),2\lambda_{\min}\left(\alpha \beta \right)-\frac{1}{\varepsilon_{2}}\lambda_{\max}\left(\alpha -K_{d}\right),\lambda_{\min}\left(K_{\zeta }\right)-\frac{1}{\varepsilon_{2}}\lambda_{\max}\left(K_{\Theta }\right)-\varepsilon_{3}\right\}$, $C=\frac{1}{2\varepsilon_{3}}{∥\Delta \tau_{i}∥}^{2}+\frac{1}{2\varepsilon_{4}}\lambda_{\max}\left({M_{i}}^{-1}\right){{\bar{d}}_{total,i}}^{2}+0.2785\lambda_{max}\left(K_{\kappa }\right)$.

## 4. Stability Analysis

**Theorem** **1.** *Considering the MSA system (1) with Assumption 1, the controller (28) and the event-triggered mechanism (30), and bounded initial conditions, where all the closed-loop signals remain bounded, the artificial potential and the tracking error can be reduced to a small area close to the origin.*

**Proof.**  Consider a Lyapunov candidate

(37) $$V=\sum_{i=1}^{n}\left(k_{a,r}P_{r,i}^{a}+\frac{1}{2}k_{a,m}P_{m,i}^{a}+\frac{1}{2}k_{r,m}P_{m,i}^{r}+\frac{1}{2}\phi k_{a,t}P_{t,i}^{a}+\frac{1}{2}\xi_{a,i}^{2}+\frac{1}{2}{\xi_{\Theta ,i}}^{T}\xi_{\Theta ,i}+\frac{1}{2}{{\hat{d}}_{i}}^{T}{\hat{d}}_{i}+\frac{1}{2}{\zeta_{i}}^{T}\zeta_{i}\right)$$

and its derivative can be obtained from (21), (23), and (36) as (38)

(38) $$\begin{array}{c} \dot{V}\leq -\sigma V+C \end{array}$$

where $\sigma =\min\{\sigma_{1},2k_{\psi },2\lambda_{\min}\left(K_{\Theta }\right)-\varepsilon_{1}\lambda_{\max}\left(K_{\Theta }\right)-\varepsilon_{2}\lambda_{\max}\left(\alpha -K_{d}\right)-\varepsilon_{4}\lambda_{\max}\left({M_{i}}^{-1}\right),2\lambda_{\min}$

$\left(\alpha \beta \right)-\frac{1}{\varepsilon_{2}}\lambda_{\max}\left(\alpha -K_{d}\right),\lambda_{\min}\left(K_{\zeta }\right)-\frac{1}{\varepsilon_{2}}\lambda_{\max}\left(K_{\Theta }\right)-\varepsilon_{3}\}$, $C=\sum_{i=1}^{n}\left(\frac{1}{2\varepsilon_{3}}{∥\Delta \tau_{i}∥}^{2}+\frac{1}{2\varepsilon_{4}}\lambda_{\max}\left({M_{i}}^{-1}\right)\right.$$\left.{{\bar{d}}_{total,i}}^{2}+0.2785\lambda_{max}\left(K_{\kappa }\right)\right)$.

Selecting suitable parameters with

$$\begin{array}{cc} \lambda_{\min}\left(K_{\Theta }\right) & \geq \frac{\varepsilon_{1}}{2}\lambda_{\max}\left(K_{\Theta }\right)+\frac{\varepsilon_{2}}{2}\lambda_{\max}\left(\alpha -K_{d}\right)+\frac{\varepsilon_{4}}{2}\lambda_{\max}\left({M_{i}}^{-1}\right)\\ \lambda_{\min}\left(\alpha \beta \right) & \geq \frac{1}{2\varepsilon_{2}}\lambda_{\max}\left(\alpha -K_{d}\right)\\ \lambda_{\min}\left(K_{\zeta }\right) & \geq \frac{1}{2\varepsilon_{1}}\lambda_{\max}\left(K_{\Theta }\right)+\frac{\varepsilon_{3}}{2}, \end{array}$$

the inequality (38) holds as

$$\begin{array}{cc} V & =\sum_{i=1}^{n}\left(k_{a,r}P_{r,i}^{a}+\frac{1}{2}k_{a,m}P_{m,i}^{a}+\frac{1}{2}k_{r,m}P_{m,i}^{r}+\frac{1}{2}\phi k_{a,t}P_{t,i}^{a}+\frac{1}{2}\xi_{a,i}^{2}+\frac{1}{2}{\xi_{\Theta ,i}}^{T}\xi_{\Theta ,i}+\frac{1}{2}{\hat{d}}_{i}^{T}{\hat{d}}_{i}+\frac{1}{2}{\zeta_{i}}^{T}\zeta_{i}\right)\\ & \leq \left(V\left(0\right)-\frac{C}{\sigma }\right)e^{-\sigma t}+\frac{C}{\sigma } \end{array}$$

The conclusions can be drawn that the tracking errors $\xi_{a,i}$, $\xi_{\Theta ,i}$ and outputs of the designed adaptive law and saturation compensator ${\hat{d}}_{i}$ and $\zeta_{i}$ are all bounded and can ultimately converge to the compact sets $\sqrt{2C/\sigma }$. The resultant ADPF is bounded as well and can be reduced to a small neighborhood a small neighborhood of zero, which can be deemed as the global minimum. Thus, the stability of the closed-loop system is proved. □

**Theorem** **2.** *The Zeno behavior is excluded in the designed event-triggered mechanism.*

**Remark** **5.** *For the designed event-triggered mechanism, Zeno behavior denotes that the designed triggered condition is constantly satisfied, which means in a finite time that there are infinitely many triggering instants. Zeno behavior is physically impossible to realize, which cannot be satisfied in practice. Moreover, Zeno behavior is against the original intention of reducing the control frequency of the actuators.*

**Proof.**  The inequality $\dot{e}\left(t\right)\leq \left|\dot{\tau_{i}}\left(t\right)\right|$ can be obtained by

(39) $$\dot{e}\left(t\right)=\dot{\tau_{i}}\left(t\right)-\dot{u_{i}}=\dot{\tau_{i}}\left(t\right)\leq \varphi$$

where φ>0.

Therefore, we obtain

(40) $$\begin{array}{cc} \int_{t_{m}}^{t_{m+1}}{\dot{e}}_{\lambda }\left(t\right)dt & =e_{\lambda }\left(t_{m+1}\right)-e_{\lambda }\left(t_{m}\right)\\ \; & =\gamma_{\lambda }\tanh\left|u_{i,\lambda }\right|+\kappa_{\lambda }-0\\ \; & \leq \varphi \left(t_{m+1}-t_{m}\right). \end{array}$$

for ∀λ=1,2,3.

It means that

(41) $$t_{m+1}-t_{m}\geq \min[(\gamma_{\lambda }\tanh|u_{i,\lambda }|+\kappa_{\lambda })/\varphi ]\geq \kappa_{\lambda }/\varphi >0$$

which reveals that the Zeno behavior is excluded because there always is a time interval between two adjacent triggered instants. □

## 5. Simulation Results

In this section, the simulation results are presented to demonstrate the effectiveness of designed method.

The main parameters of the stratospheric airship model are presented in Table 1. More practically, the limits of control forces and control torque are given in Table 2.

The target region is defined as a circular area whose center coordinate is [0 km, 0 km] and radius is 50 km. The coverage mission is to be performed by an MSA system consisting of seven stratospheric airships. The initial positions of the MSA system are set as $\chi_{1}\left(0\right)={\left[-101\;\mathrm{km},-101.3\;\mathrm{km}\right]}^{T}$, $\chi_{2}\left(0\right)={\left[-101\;\mathrm{km},-101.6\;\mathrm{km}\right]}^{T}$, $\chi_{3}\left(0\right)=[-101\;\mathrm{km}$, ${-101.9\;\mathrm{km}]}^{T}$, $\chi_{4}\left(0\right)\;=\;{\left[-101\;\mathrm{km},-151.2\;\mathrm{km}\right]}^{T}$, $\chi_{5}\left(0\right)={\left[-101.3\;\mathrm{km},-101\;\mathrm{km}\right]}^{T}$, $\chi_{6}\left(0\right)=$ [−101.6km, ${-101\;\mathrm{km}]}^{T}$, and $\chi_{7}\left(0\right)={\left[-101.9\;\mathrm{km},-101\;\mathrm{km}\right]}^{T}$, and the initial yaw angles of the MSA system are set as $\psi_{1}\left(0\right)\;=\;0$, $\psi_{2}\left(0\right)\;=\;0$, $\psi_{3}\left(0\right)\;=\;0$, $\psi_{4}\left(0\right)\;=\;0$, $\psi_{5}\left(0\right)\;=\;90$, $\psi_{6}\left(0\right)\;=\;90$, and $\psi_{7}\left(0\right)\;=\;90$. The coverage zone of a stratospheric airship is assumed to be the circle with its center at the stratospheric airship’s position, and the radius is 50km. The coverage area of the target region is mathematically matched to the total coverage ability of the MSA system. The external disturbances are set as $d_{i}=[400+200d_{i,r}\left(N\right),50+100d_{i,r}\left(N\right),$1000+1000$d_{i,r}\left(N\cdot m\right){]}^{T}$ with $d_{i,r}=0.3\sin\left(t/120\right)+0.5\cos\left(t/360\right)$. The sampling period for monitoring the mixed-triggering conditions is 0.1 s.

The control parameters for the virtual control law are chosen as $k_{p}=0.3$, $k_{r,a}=1\times 10^{-6}$, $k_{m,r}=2\times 10^{9}$, $k_{m,a}=2\times 10^{-1}$, $k_{t,a}=2\times 10^{-4}$, $k_{\psi }=2$, $\eta_{a,m}=0.3$, and $\eta_{r,m}=0.3$, $\eta_{a,t}=0.3$. The control parameters for the event-triggered adaptive tracking controller are chosen as $\alpha =\mathrm{diag}\left\{1,1,100\right\}\times 10^{-3}$, β=diag{100,100,1}, $K_{\Theta }=\mathrm{diag}\left\{2,0.05,0.2\right\}\times 10^{-3}$, $K_{d}=\mathrm{diag}\left\{1,1,100\right\}$, $K_{\zeta }=\mathrm{diag}\left\{3,3,60\right\}\times 10^{-2}$, $\bar{\kappa }=\mathrm{diag}\left\{400,600,3000\right\}$, γ=diag{40,60,400}, and κ=diag{200,400,2000}.

**Figure 5:**
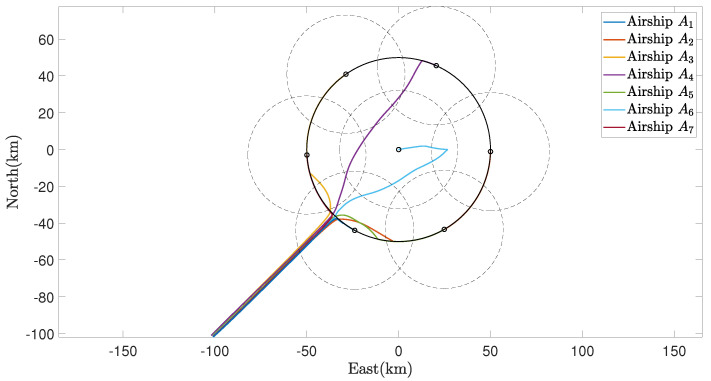
Trajectories of the MSAs system in the region coverage process.

**Figure 6:**
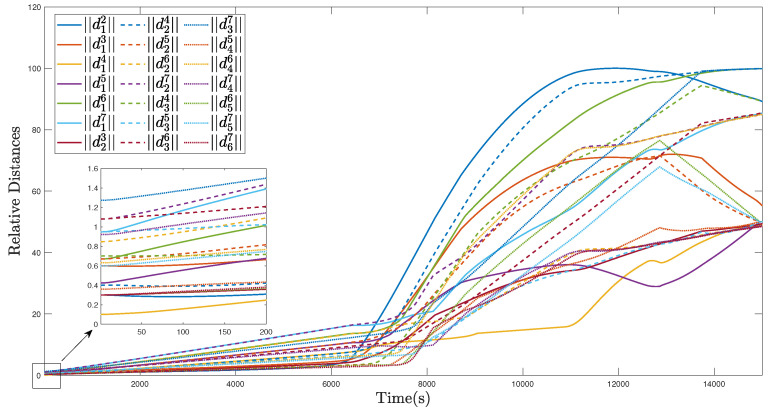
Relative distances of the MSAs system in the region coverage process.

The trajectories of the MSA system in the region coverage process are given in Figure 5. As illustrated, the MSA system can be guided and controlled to fly to the target region under the effect of the defined region attractive potential function $P_{a,r}$, after which, each member of the MSA system can be deployed to fully cover the target region with $P_{r,m}$. In this process, the relative distances are given in Figure 6, and all the relative distances can converge to several fixed values, indicating that the members of the MSAs are properly deployed according to the defined mutual repulsive potential function $P_{r,m}$. In addition, no collisions occur.

**Figure 7:**
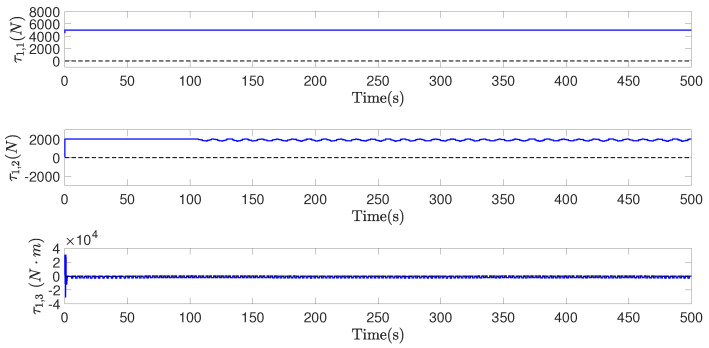
$\tau_{1}$, control inputs of airship $A_{1}$, in former 500 s.

**Figure 8:**
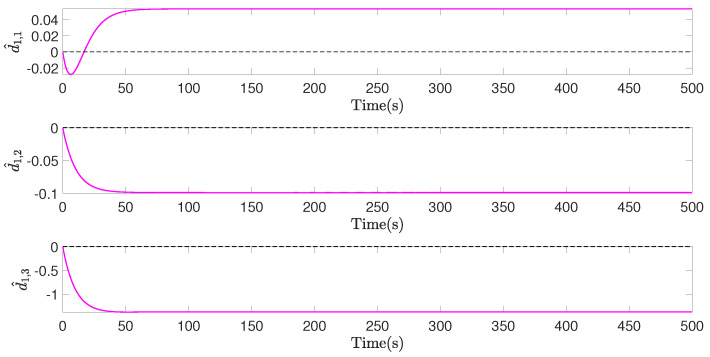
${\hat{d}}_{1}$, outputs of adaptive law of airship $A_{1}$, in former 500 s.

**Figure 9:**
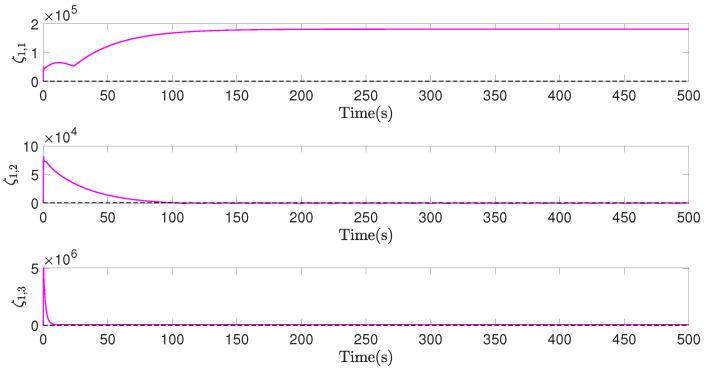
$\zeta_{1}$, outputs of saturation compensator of airship $A_{1}$, in former 500 s.

Figure 7, Figure 8 and Figure 9, respectively, show the control inputs, the outputs of the adaptive law, and the outputs of the saturation compensator of airship $A_{1}$, in a period of the former 500 seconds, as an example. Figure 7 demonstrates the control inputs under the saturation limitation, and no oscillations exists. Moreover, the control inputs are discretized by the effect of the designed event-triggered mechanism, significantly reducing the control frequency of the actuator. Figure 8 and Figure 9 illustrate that the outputs of the adaptive law and the saturation compensator all can be converged, so all of the signals of the closed-loop system are bounded.

**Figure 10:**
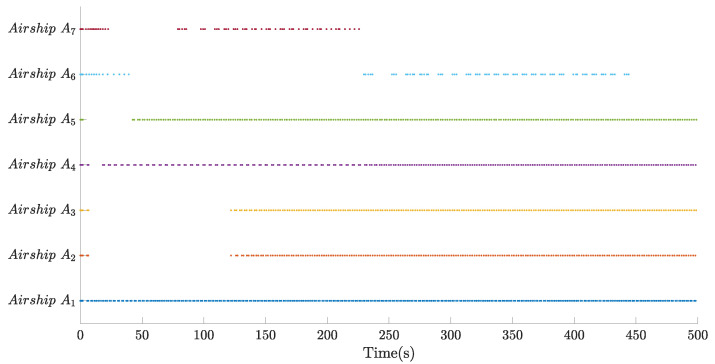
Event-triggered instants in former 500 s.

**Figure 11:**
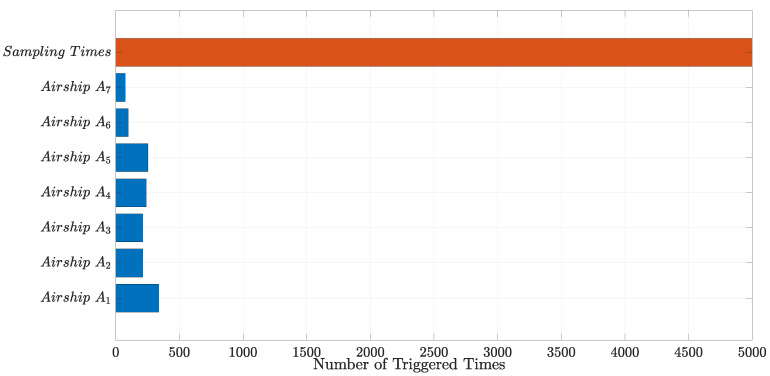
Triggered times constrast between the event-triggered mechanism and the time-triggered mechanism.

Moreover, some statistical analysis results about the designed event-triggered mechanism are given in Figure 10 and Figure 11, from which we can know that more than 90% of the triggered time is saved compared with the traditional time-triggered mechanism.

**Figure 12:**
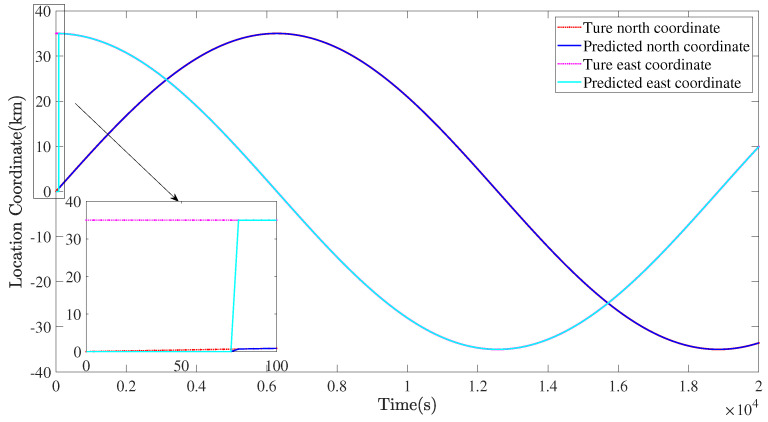
Prediction results of location information.

**Figure 13:**
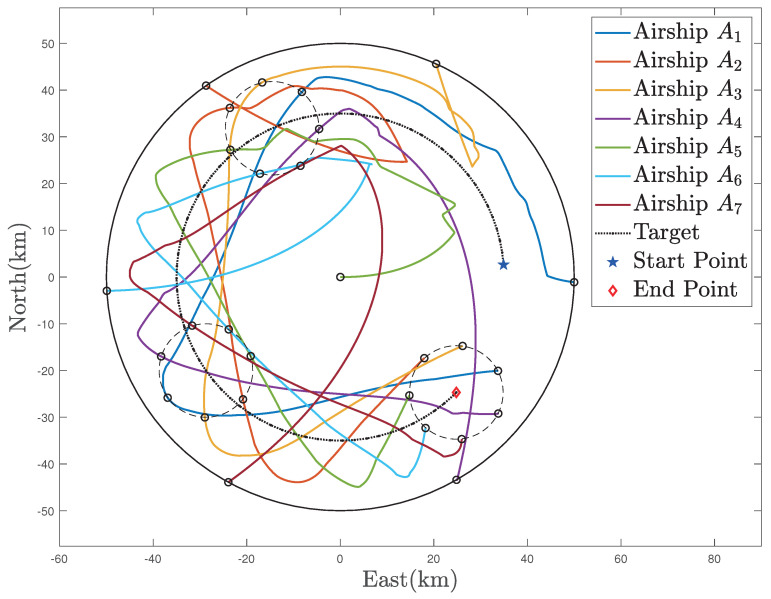
Trajectories of the MSAs system in the moving target coverage process.

**Figure 14:**
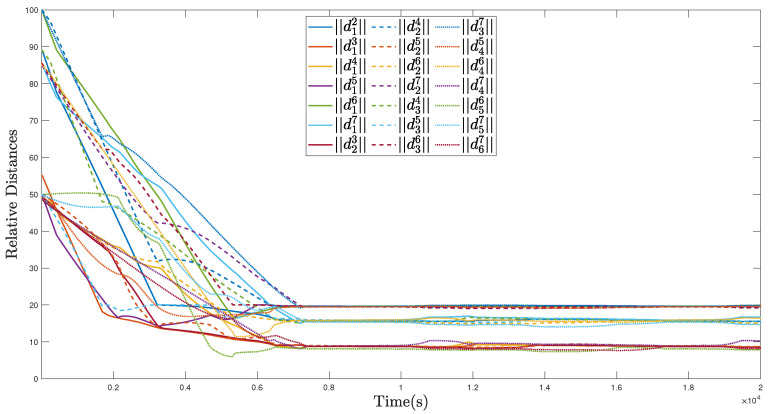
Relative distances of the MSAs system in the moving target coverage process.

**Figure 15:**
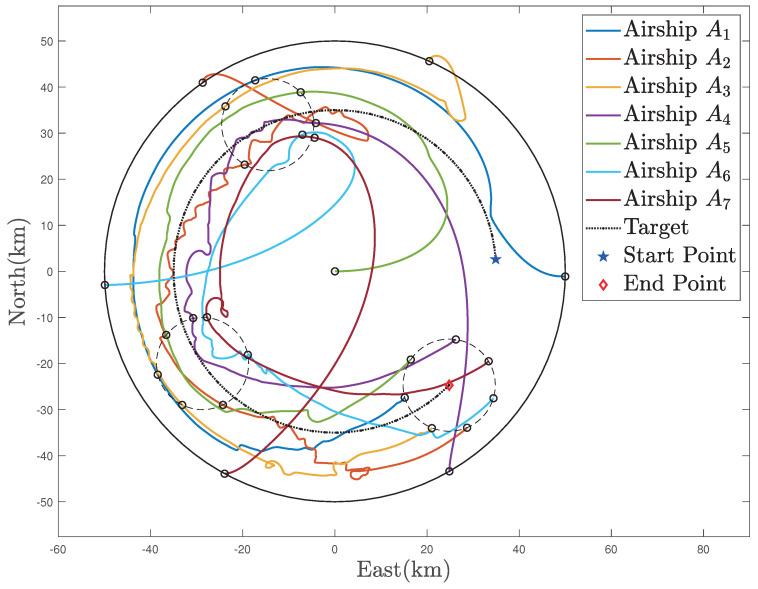
Trajectories of the MSAs system in the moving target coverage process with traditional APF-based method.

**Figure 16:**
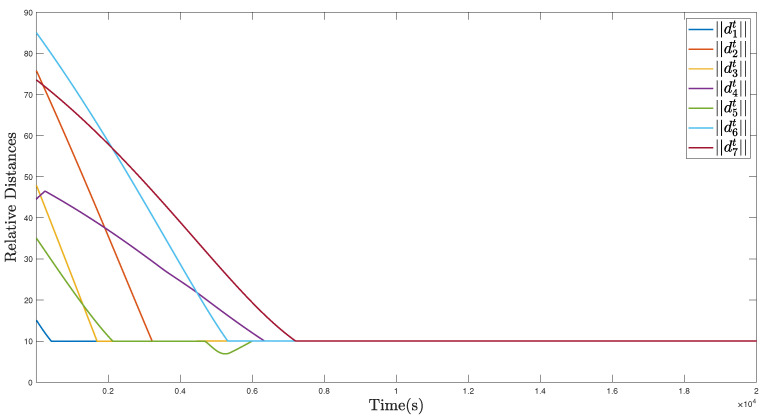
Relative distances between the MSAs system members and the moving target.

In Figure 12, Figure 13 and Figure 14, the simulation results of the MSA system’s tight coverage state for the detected target are given. First, the prediction results of the AR model are displayed in Figure 12, in which the prediction begins at 75 s and, afterwards, the predicted values of the location remain the same with their true values. As illustrated in Figure 13, the MSA system can be guided and controlled to tightly cover this moving target under the effect of the defined moving target attractive potential function $P_{a,t}$. In Figure 13 and Figure 15, the circles denote the stratospheric airships. At the same time, as shown in Figure 14, we can know that all the relative distances can converge to several fixed values, and no collisions occur, from Figure 16. Moreover, all of the relative distances between the MSA system members and the moving target can all converge to the radius of the moving target to realize the moving target coverage.

For comparison, the simulation result of the proposed control framework with the traditional APF method is given, in which the obvious oscillations occur due to the absence of the adaptive modification in ADPF.

## 6. Conclusions

In this paper, we proposed a dynamic coverage control framework using the MSAs system, with several practical application problems, such as the processing delay, external disturbances, and input saturation. The dynamic coverage mission can be completed, so that the target region can be covered, and the coverage target can be switched to the monitored or observed moving target. The autoregressive model was introduced to effectively predict the location information to eliminate the influence of processing delay. The potential field-based virtual control law can guide MSAs system members to be deployed to cover the target region or moving target, in which the APF is improved to an ADPF to modify the generated potential field to adapt to the dynamic scenarios. To address the external disturbances and nonsmooth input saturation problem in the backstepping design architecture, the adaptive law and the saturation compensator were designed. At the same time, to prolong the actuator life, the control frequency was significantly reduced by the effect of the designed event-triggered mechanism. The Lyapunov stability and Zeno behavior exclusion analyses were given mathematically. The simulation results illustrated the effectiveness of the proposed dynamic coverage control framework. In future studies, we plan to conduct a high-altitude flight test to evaluate the capability of the proposed control framework in reality. Moreover, more application problems such as coupling and communication delay will be addressed in our future work. 

## Figures and Tables

**Table 1:** Model Parameters.

Parameter	Value	Units
*m*	$4.2\times 10^{3}$	kg
∇	$3.2\times 10^{4}$	m${}^{3}$
*g*	9.8	m/s${}^{2}$
$\left[I_{x},I_{y},I_{xy}\right]$	$\left[4,25,1\right]\times 10^{5}$	kg·m${}^{2}$
$\left[k_{1},k_{2},k_{3}\right]$	[0.1,0.1,400]	

**Table 2:** Limits of Control Forces and Control Torque.

Forces & Torque	Value	Units
Force $F_{x}$	[0,5000]	N
Force $F_{y}$	[−2000,2000]	N
Torque $M_{z}$	[−50,000,50,000]	N·m${}^{2}$

## Data Availability

The data that support the findings of this study are available from the corresponding author upon reasonable request.
